# CCAAT/Enhancer-Binding Protein Alpha Is a Novel Regulator of Vascular Smooth Muscle Cell Osteochondrogenic Transition and Vascular Calcification

**DOI:** 10.3389/fphys.2022.755371

**Published:** 2022-02-28

**Authors:** Pengyuan Chen, Wanzi Hong, Ziying Chen, Flora Gordillo-Martinez, Siying Wang, Hualin Fan, Yuanhui Liu, Yining Dai, Bo Wang, Lei Jiang, Hongjiao Yu, PengCheng He

**Affiliations:** ^1^Department of Cardiology, Guangdong Provincial People’s Hospital’s Nanhai Hospital, The Second Hospital of Nanhai District Foshan City, Foshan, China; ^2^Institute of Cardiovascular Disease, Guangdong Key Laboratory of Vascular Diseases, State Key Laboratory of Respiratory Disease, The Second Affiliated Hospital, Guangzhou Medical University, Guangzhou, China; ^3^School of Medicine, Guangdong Provincial People’s Hospital, South China University of Technology, Guangzhou, China; ^4^Department of Pathology, The Sixth Affiliated Hospital of Guangzhou Medical University, Qingyuan People’s Hospital, Qingyuan, China; ^5^Institute of Science and Environment, University of Saint Joseph, Macao, Macao SAR, China; ^6^Department of Cardiology, Guangdong Cardiovascular Institute, Guangdong Provincial Key Laboratory of Coronary Heart Disease Prevention, Guangdong Provincial People’s Hospital, Guangdong Academy of Medical Sciences, Guangzhou, China; ^7^Department of Biochemistry and Molecular Biology, Guangzhou Medical University-Guangzhou Institutes of Biomedicine and Health Joint School of Life Sciences, Guangzhou Medical University, Guangzhou, China; ^8^School of Medicine, The Second School of Clinical Medicine, Southern Medical University, Guangzhou, China

**Keywords:** vascular calcification, CCAAT/enhancer-binding protein alpha, vascular smooth muscle cells, osteogenic differentiation, calcium deposition

## Abstract

**Aims:**

Vascular calcification is a common clinical complication of chronic kidney disease (CKD), atherosclerosis (AS), and diabetes, which is associated with increased cardiovascular morbidity and mortality in patients. The transdifferentiation of vascular smooth muscle cells (VSMCs) to an osteochondrogenic phenotype is a crucial step during vascular calcification. The transcription factor CCAAT/enhancer-binding protein alpha (C/EBPα) plays an important role in regulating cell proliferation and differentiation, but whether it regulates the calcification of arteries and VSMCs remains unclear. Therefore, this study aims to understand the role of C/EBPα in the regulation of vascular calcification.

**Methods and Results:**

Both mRNA and protein expression levels of C/EBPα were significantly increased in calcified arteries from mice treated with a high dose of vitamin D3 (vD3). Upregulation of C/EBPα was also observed in the high phosphate- and calcium-induced VSMC calcification process. The siRNA-mediated knockdown of C/EBPα significantly attenuated VSMC calcification *in vitro*. Moreover, C/EBPα depletion in VSMCs significantly reduced the mRNA expression of the osteochondrogenic genes, e.g., sex-determining region Y-box 9 (Sox9). C/EBPα overexpression can induce SOX9 overexpression. Similar changes in the protein expression of SOX9 were also observed in VSMCs after C/EBPα depletion or overexpression. In addition, silencing of Sox9 expression significantly inhibited the phosphate- and calcium-induced VSMC calcification *in vitro*.

**Conclusion:**

Findings in this study indicate that C/EBPα is a key regulator of the osteochondrogenic transdifferentiation of VSMCs and vascular calcification, which may represent a novel therapeutic target for vascular calcification.

## Introduction

Cardiovascular disease is the leading cause of death globally, and vascular calcification is the basic pathologic change. Previous studies have demonstrated that both chronic kidney disease (CKD) and diabetes are the independent factors for predicting cardiovascular events (Moe and Chen, 2004). A possible explanation is that vascular calcification is prevalent in CKD or diabetes. The initialization of vascular calcification shares the activation of oxidative stress, inflammation, and mineral metabolic disorder, and the mineral deposition in the medial artery is thought to be the key part (Yahagi et al., 2017).

Vascular calcification is a pathologic change of the vascular wall resulting from mineral deposition which can increase the risk of cardiovascular disease (CVD), stroke, and atherosclerosis (AS) (Nicoll and Henein, 2014). It has been reported that vascular calcification is significantly associated with the imbalanced mineral metabolism in the human body (Paloian and Giachelli, 2014; Yamada and Giachelli, 2017). Besides, the change from contractile to chondrogenic phenotype of vascular smooth muscle cells (VSMCs) is known to play a key role in vascular calcification. In response to vascular plasticity, VSMCs are characterized by the expression of SMC-specific contractile proteins (Sinha et al., 2014). Similar to osteogenic differentiation of bone, vascular calcification is characterized by some key osteogenic regulators. Typically, the osteogenic transformation of VSMCs is the characteristic change during vascular calcification process, with decreased expression of contractile proteins like SMA, whereas increased expression of osteogenic genes, such as Runt-related transcription factor 2 (Runx2), bone morphogenetic protein-2 (Bmp2), osteopontin (OPN), alkaline phosphatase (ALP), and sex-determining region Y-box 9 (Sox9) (Shanahan et al., 2011).

The CCAAT/enhancer-binding protein alpha (C/EBPα) belongs to the family of C/EBP-homologous protein (CHOP), which is known to trigger the transformation of adipocyte phenotype (Tang and Lane, 2012). Although it has been previously reported that adipocyte accelerates the tissue calcification, the role of adipocyte transformation in vascular calcification remains obscure (Chen et al., 2014). Recently, researchers have demonstrated the role of CHOP in AS and valve calcification (Zhou et al., 2015). It has also been reported that C/EBPα regulates osteogenic genes, including bone morphogenetic protein-2 (BMP2) and SOX9 (Ichida et al., 2004; Fan et al., 2009; Casado-Diaz et al., 2016). Theoretically, C/EBPα can promote vascular calcification by the overexpression of osteogenic genes.

In this study, we observed the upregulation of C/EBPα in tissue vascular calcification. Furthermore, we revealed the activation of C/EBPα in the calcification medium of primary mouse VSMC. Calcification activated the expression of C/EBPα and, subsequently, induced the expression of osteogenic genes. Collectively, this study indicates the positive effect of C/EBPα on vascular calcification both *in vitro* and *in vivo*, which may become a potential therapeutic target for vascular calcification in the future.

## Materials and Methods

### Materials

All animal experiments were approved by the Experimental Animal Ethics Committee of Guangzhou Medical University. C57BL/6 male mice were purchased from Dien Gene Com. (Guangzhou, China) and maintained in accordance with the guidelines for the care and use of laboratory animals of Guangzhou Medical University. Trypsin for the isolation of VSMCs was purchased from Gibco (Carlsbad, CA, United States) (Cat# 12605-010). Na_2_HPO_4_/NaH_2_PO_4_ (Pi) (Cat# RES20908/RDD007), calcium chloride (CaCl_2_) (Cat# 5670-100G), and vitamin D3 (vD3; cholecalciferol, Cat# 47763) were obtained from sigma to prepare the calcium culture medium and animal model reagents. The basic cell culture medium consisted of Minimum Essential Medium α (α-MEM) supplemented with 10% Fetal Bovine Serum (FBS) (Gibco, Cat# 16000-044), 100 U/ml penicillin (HyClone, Bath, United Kingdom, Cat# SH40003.01), and 100 mg/ml streptomycin (HyClone, Cat# SV30010).

### Isolation of Primary Mouse VSMCs

The primary mouse VSMCs were isolated as described in the previous study (He et al., 2020). Briefly, the descending aorta was isolated from the 6-week-old male mice. Thereafter, the inner and outer layers of the vessel were removed by trypsin or microscissors. Arteries were then digested in 425 U/ml collagenase type II (Worthington, Cergy Pontoise Cedex France, Cat# 47D17411A) for 5 h at 37°C. Later, the cells obtained were resuspended in the basic culture medium. Then, VSMCs were seeded in a 25 cm^2^ flask coated with 0.25 μg/cm^2^ type I collagen (Gibco, Cat# A1048301). The VSMCs were verified by immunofluorescence, in which the smooth muscle marker was stained. The isolation of VSMCs was successful to reach 90% in cells (Supplementary Figure 1). Cells in the second passage were harvested for experiments.

### Induction of Calcification in Primary Mouse VSMCs

Primary mouse VSMCs were incubated with control (1.0 mM Pi/1.8 mM Ca) or calcification medium (50 μg/ml ascorbic acid/2.5 mM Pi/2.7 mM Ca) for up to 7 days. Later, 1 M phosphate was prepared with Na_2_HPO_4_/NaH_2_PO_4_ at a weight ratio of 55:14. The cell medium was changed every 3 days. Meanwhile, calcium deposition was determined by alizarin red staining, as described in the previous study. Briefly, VSMCs were fixed with 4% paraformaldehyde (PFA) at room temperature for 15 min and, subsequently, stained with 2% alizarin red (pH 4.2) for 10 min at room temperature. The tissue calcification areas were normalized to the vascular circumference, and all the semi-quantification results of primary mouse VSMCs staining were available in the Supplementary Figure 5.

### Murine Model of Vascular Calcification

The induction of murine vascular calcification was performed as previously described (He et al., 2020). Briefly, 6-week-old C57BL/6 male mice were given, subcutaneously injected of 5 × 10^5^ IU/kg vD3 or vehicle once a day for 3 days, and sacrificed at 7 days after injection. The descending aorta was isolated after removing adipocytes. Tissues were fixed with PFA for further staining or filmmaking and then frozen at −20°C for further RNA or protein analysis.

### Analysis of Calcification

The VSMCs seeded in six-well plates were washed twice with phosphate buffered solution (PBS) and decalcified with 0.6 M hydrochloric acid (HCL) for 24 h. Later, calcium was quantified by colorimetric assay (Sigma, Taufkirchen Germany, Cat# MAK022-1KT) according to the instructions of the manufacturer. Then, decalcified cells were harvested with 0.1 M sodium hydroxide (NaOH) supplemented with 0.1% Sodium Dodecyl Sulfate (SDS). The calcium quantification (μg) was normalized to protein (mg).

### RT-PCR

Total RNA was extracted using the SteadyPure Universal RNA Extraction Kit (Accurate Biotechnology Co., Ltd., Hunan, China; Cat# AG21017) in line with the instructions of the manufacturer. Later, the extracted total RNA was quantified and prepared into cDNA through reverse transcription using the Evo M-MLV RT Premix for qPCR (Accurate Biotechnology Co., Ltd., Hunan, China; Cat# AG11706). RT-qPCR was later performed using the QuantStudio 5 Real-Time System (Life Technologies) with SYBR Green Premix Pro Taq HS qPCR Kit (Accurate Biotechnology Co., Ltd., Hunan, China; Cat# AG11701). Each PCR procedure was run in duplicate. All gene expression data were calculated using the 2^–ΔΔCT^ method and normalized to β-ACTIN. The primer sequences for target genes are summarized in Supplementary Table 1.

### Western Blotting Analysis

The VSMCs and murine tissues were harvested with RIPA lysis buffer (Beyotime Biotechnology, Shanghai, China, Cat# P0013B) supplemented with 1 mM protease inhibitor phenylmethylsulfonyl fluoride (PMSF) (Beyotime Biotechnology, Cat# ST505). Therefore, the total protein was quantified using Micro BCATM Protein Assay Kit (Thermo Fisher Scientific, Waltham, MA, United States, Cat# 23235). An equal amount of proteins were separated by SDS-PAGE and transferred to the polyvinylidene difluoride (PVDF) membranes. Later, the membranes were incubated overnight at 4°C with the following primary antibodies: anti-BMP2 (1:2,000; Abcam, Waltham, MA, United States, Cat# ab214821, RRID:AB_2814695), anti-C/ebpα (1:2,000; Santa Cruz Biotechnology, Dallas, TX, United States, Cat# sc-166258, RRID:AB_2078042), anti-Flag (1:2,000; Proteintech, Rosemont, IL, United States, Cat# 66008-3-Ig, RRID:AB_2749837), anti-OPN (1:2,000; Proteintech, Cat# 22952-1-AP, RRID:AB_2783651), anti-SOX9 (1:2,000; Cell Signaling Technology, Danfoss, MA, United States, Cat# 82630, RRID:AB_2665492), anti-α-actin (1:2000; Santa Cruz Biotechnology, Cat# sc-56499, RRID:AB_830982), anti-β-actin (1:2,000; Santa Cruz Biotechnology, Cat# sc-81178, RRID:AB_2223230), and anti-GAPDH (1:2,000; Santa Cruz Biotechnology, Cat# sc-365062, RRID:AB_10847862). Subsequently, the membranes were further incubated with horseradish peroxidase (HRP)-conjugated anti-mouse (1:4,000; Cell Signaling Technology, Cat# 7076S) or anti-rabbit (1:4,000; Cell Signaling Technology, Cat# 7074S) secondary antibody for 1 h at room temperature. The immune complexes were visualized by chemiluminescence, i.e., Lumi-Light Western Blotting (WB) Substrate (Millipore, Cat# WBKLS0500). The ImageJ software (the National Institutes of Health) was employed for the semiquantitative assessment of band intensity.

### siRNA Transfection

The VSMCs were seeded at the density of 1.0 × 10^5^ cells/well in 6-well plates and transfected with 25 nM c/ebpα siRNA or scrambled siRNA (RIBOBIO, Guangzhou, China) by using Lipofectamine RNAiMAX (Invitrogen, Waltham, MA, United States, Cat# 13778), following the instructions of the manufacturer. The siRNA silencing efficiency was verified by RT-qPCR and WB assays. Cell transfection was conducted every 3 days. The siRNA sequences for gene silencing are listed in the Supplementary Material.

### Adenovirus-Mediated Overexpression of c/ebpα

Recombinant adenovirus vectors expressing c/ebpα (Ad-c/ebpα) gene or recombinant adenovirus carrying green fluorescent protein (Ad-GFP) gene were purchased from Hanheng Bioscience Incorporation, Shanghai, China. Primary mouse VSMCs were seeded at the density of 1.0 × 10^5^ cells/well in six-well plates. After 85% confluence, cells were incubated with Ad-c/ebpα or Ad-GFP at an multiple of infection (MOI) of 50 for every 3 days. The c/ebpα overexpression efficiency was confirmed by RT-qPCR and WB assays.

### Statistical Analysis

All data were expressed as mean ± SEM. Statistical analysis was performed using the GraphPad Prism 6 (La Jolla, CA, United States) software. The Shapiro–Wilk test was adopted to test the data normality. Data between two groups were compared using unpaired Student’s *t*-test, while those among multiple groups were compared by one-way ANOVA followed by the Bonferroni *post hoc* test or a suitable non-parametric test, such as the Mann–Whitney *U* test. *P* < 0.05 was considered to be statistically significant.

## Results

### CCAAT/Enhancer-Binding Protein Alpha Was Upregulated During Vascular Calcification *in vivo*

**FIGURE 1:**
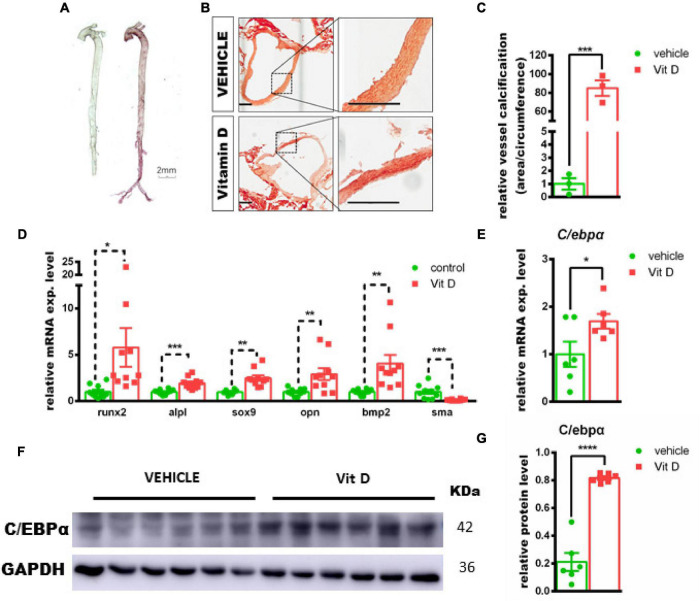
Association of CCAAT/enhancer-binding protein alpha (c/ebpα) with vascular calcification in the murine vascular calcification model. **(A)** Alizarin red staining for calcium deposition in aorta from vitamin D (vD)-treated mice. Scale bar, 2 mm. **(B)** Typical images at 100× magnification of aorta paraffin section from vD-treated mice or vehicle-treated mice stained by alizarin red; the indicated areas are enlarged by 4-fold and placed beside the respective panels. Scale bar, 200 μm. **(C)** Semiquantitative analysis of vascular wall calcification presented at area to circumference ratio (*n* = 3). **(D–E)** Real-time PCR for mRNA expression of Runt-related transcription factor 2 (Runx2), Alpl, sex-determining region Y-box 9 (Sox9), bone morphogenetic protein-2 (Bmp2), osteopontin (Opn), Sma, and c/ebpα in vascular calcified aortas for indicated experimental groups. The target mRNAs were normalized to **β***-actin* mRNA and are graphed (*n* = 8). **(F)** Representative images of Western blotting for C/EBPα in vascular calcification aortas from vD-treated mice and vehicle controls. **(G)** Semiquantitative analysis of Western blotting for C/EBPα protein expression using ImageJ software (*n* = 6). *p < 0.05, **p < 0.01, ***p < 0.001, ****p < 0.0001.

First of all, we validated the vitamin D-induced mouse vascular calcification model *in vivo*. In accordance with the previous study (He et al., 2020), vitamin D injection increased calcium deposition in murine aorta, as determined by alizarin red staining (Figures 1A–C). In addition, the critical regulators of osteogenic differentiation, such as *Runx2 and Bmp2*, were significantly upregulated in calcified aortic tissues following vitamin D treatment at day 7, while the smooth muscle contractile marker, SMA, was significantly downregulated (Figure 1D). The mRNA expression genes, such as *Runx2* (5.7-fold, *p* = 0.035), *Alpl* (1.9-fold, *p* < 0.001), *Opn* (2.9-fold, *p* < 0.01), *Bmp2* (4.0-fold, *p* < 0.01), and *Sox9* (2.4-fold, *p* < 0.01), were significantly increased in vascular calcification murine model, while the mRNA expression of *Sma* was significantly decreased by 90% (*p* < 0.01). Compared with vehicle tissues, both C/ebpα mRNA and protein expression were significantly upregulated in calcified arteries isolated from vD-treated mice (Figures 1E–G). Taken together, these data suggested that vitamin D-induced murine vascular calcification was associated with an osteogenic phenotype, and C/EBPα expression was upregulated during vascular calcification of tissues.

### CCAAT/Enhancer-Binding Protein Alpha Was Upregulated in Calcium-/Phosphate-Induced Primary Mouse VSMC Calcification *in vitro*

**FIGURE 2:**
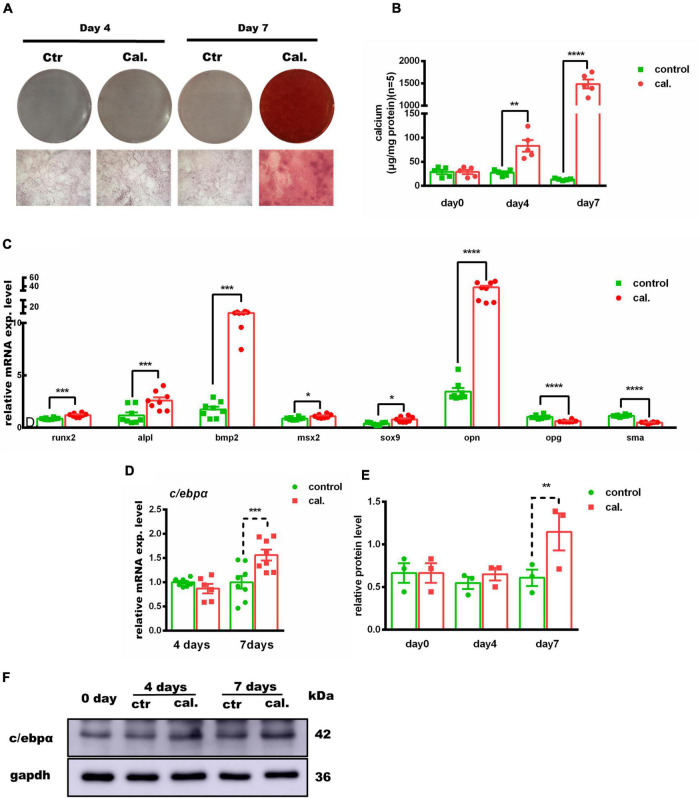
Association of c/ebpα with vascular calcification in the primary vascular smooth muscle cell (VSMC) vascular calcification model. **(A)** Representative images of alizarin red staining confirmed calcium deposition in VSMCs treated with calcium/phosphate or control medium. Typical areas at 10× magnifications are placed beside the respective panels. **(B)** Quantitative calcium assay for VSMCs treated with calcium/phosphate or control medium in different days (*n* = 5). **(C,D)** Real-time PCR analysis for the mRNA expression of *Runx2*, *Alpl*, *Bmp2*, *Msx2*, *Sox9*, *Opn*, *Opg*, *Sma*, and *C/ebp*α in VSMCs vascular calcification for indicated experimental groups. The target mRNAs were normalized to β*-actin* mRNA and are graphed (*n* = 8). **(E)** Semiquantitative analysis of Western blotting for C/EBPα protein expression using ImageJ software (*n* = 3). **(F)** Representative images of Western blotting of VSMCs vascular calcification for C/EBPα protein expression. *p < 0.05, **p < 0.01, ***p < 0.001, ****p < 0.0001.

Second, we validated the calcium-/phosphate-induced primary VSMC calcification model *in vitro*. The calcium deposition was significantly increased at day 4 and day 7 in calcium/phosphate treatment groups (day 4: *p* < 0.01; day 7: *p* < 0.0001) (Figures 2A–C and Supplementary Figure 2). In addition, the mRNA expression of *Opn* was significantly increased (2.2-fold increased, *p* < 0.0001), while *Sma* was significantly downregulated (−30%, *p* < 0.0001) at day 4. The mRNA expression of *Opg* and *Sma* was dramatically downregulated (*Opg*: −40%, *p* < 0.0001; *Sma*: −58%, *p* < 0.0001), and that of osteogenic genes, such as *Runx2*, *Alpl, Bmp2*, *Msx2*, *Sox9*, and *Opn*, was evidently upregulated at day 7 (*Runx2*: 1.39-fold, *p* < 0.0001; *Alpl:* 2.22-fold, *p* < 0.001; *Bmp2*: 6.07-fold, *p* < 0.0001; *Msx2*: 1.25-fold, *p* < 0.05; *Sox9*: 2.02-fold, *p* < 0.05; *Opn*: 10.9-fold, *p* < 0.0001). Both C/ebpα mRNA and protein levels were remarkably upregulated in the calcified primary VSMCs (Figures 2D–F). Taken together, these data revealed that the calcium-/phosphate-induced primary VSMC calcification was associated with an osteogenic phenotype, and C/ebpα was upregulated.

### Knockdown of C/ebpα Decreased Primary VSMC Calcification Induced by Calcium/Phosphate *in vitro*

**FIGURE 3:**
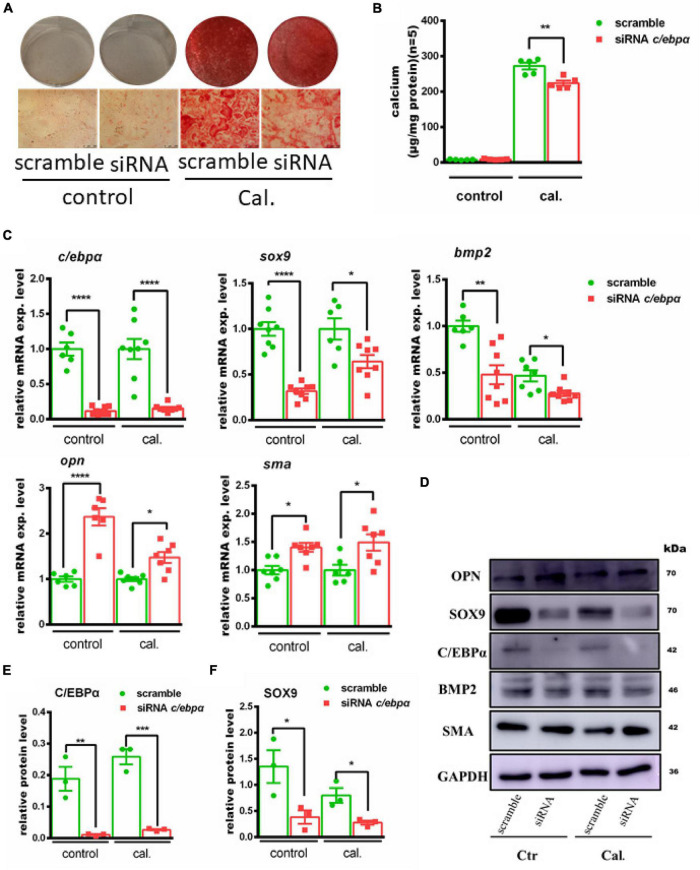
Silencing the c/ebpα inhibits calcium-/phosphate-induced vascular calcification. **(A)** Representative images of alizarin red staining confirmed calcium deposition in primary VSMCs transfected with 25 nM scrambled or siRNA c/ebpα treated with calcium/phosphate or control medium. Typical areas at 10 × magnifications are placed beside the respective panels. **(B)** Quantitative calcium assay for VSMCs transfected with 25 nM scrambled or siRNA c/ebpα treated with calcium/phosphate or control medium (*n* = 5). **(C)** Real-time PCR analysis for the mRNA expression of *Bmp2*, *Sox9*, *Opn*, *Opg*, *Sma*, and *c/ebp*α in VSMCs vascular calcification for the indicated experimental groups. The target mRNAs were normalized to β*-actin* mRNA and are graphed (*n* = 8). **(D)** Western blotting analysis of VSMCs vascular calcification for the indicated proteins. Indicated protein expression was normalized to GAPDH. **(E,F)** Semiquantitative analysis of Western blotting for C/EBPα and SOX9 protein expression using ImageJ software (*n* = 3). *p < 0.05, **p < 0.01, ***p < 0.001, ****p < 0.0001.

The functional role of C/ebpα in calcium-/phosphate-induced primary VSMC calcification was further investigated. Transfection of C/ebpα siRNA resulted in a remarkable downregulation of C/ebpα in both mRNA (Figure 3C) and protein levels (Figure 3D). Silencing of C/ebpα significantly decreased calcium-/phosphate-induced primary VSMC calcification *in vitro*, as confirmed by alizarin red staining and calcium quantitative assay (Figures 3A,B). Simultaneously, mRNA expression of osteogenic genes, such as *Alpl*, *Bmp2*, and *Sox9*, was decreased, while mRNA expression of contractile genes, such as *Opn* and *Sma*, was increased after siRNA C/ebpα treatment (Figures 3C,D). The semi-quantification also confirmed the similar change trends to mRNA of C/EBPα and SOX9 (Figures 3E,F). These data demonstrated that C/ebpα is a novel enhancer of vascular calcification.

### Overexpression of C/ebpα Increased Primary VSMC Calcification Induced by Calcium/Phosphate *in vitro*

**FIGURE 4:**
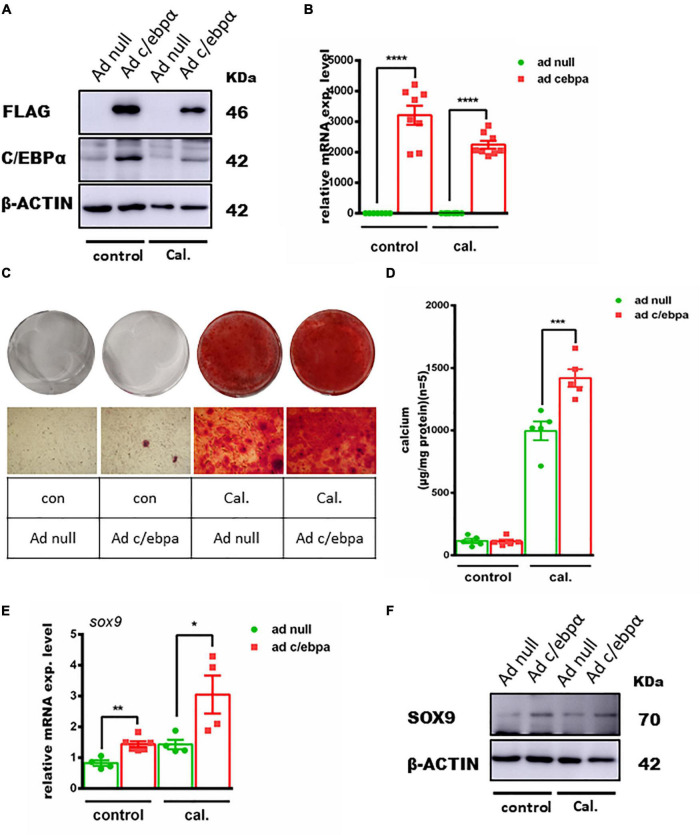
Overexpression of the c/ebpα enhanced calcium-/phosphate-induced VC through upregulating SOX9. **(A)** Western blotting of VSMCs incubated with Ad-null or Ad-c/ebpα at an MOI of 50 treated with calcium/phosphate or control medium for the c/ebpα protein. **(B)** Real-time PCR analysis for *c/ebp*α mRNA expression in VSMCs for the indicated experimental groups. The target mRNAs were normalized to β*-actin* mRNA and are graphed (*n* = 8). **(C)** Typical images of alizarin red staining confirmed calcium deposition in VSMCs for the indicated experimental groups. Typical areas at 10 × magnifications are placed beside the respective panels. **(D)** Quantitative calcium assay for VSMCs for the indicated experimental groups (*n* = 5). **(E)** Real-time PCR analysis for *sox9* mRNA expression in VSMCs for the indicated experimental groups. The target mRNAs were normalized to β*-actin* mRNA and are graphed (*n* = 4–8). **(F)** Western blotting of SOX9 in VSMCs for the indicated experimental groups. *p < 0.05, **p < 0.01, ***p < 0.001, ****p < 0.0001.

To further confirm the effect of C/ebpα on VSMC calcification, we overexpressed C/ebpα in VSMCs using adenovirus (Figures 4A,B). Alizarin red staining and calcium quantitative analysis showed that Ad-C/ebpα significantly increased calcium deposition in VSMCs treated with calcium/phosphate (Figures 4C,D). The qPCR and WB assay showed that SOX9 was also increased after overexpressing C/ebpα (Figures 4E,F). Therefore, the results showed that C/ebpα promotes calcium-/phosphate-induced VSMC calcification by upregulating *Sox9*.

### Knockdown of *sox9* Decreased VSMC Calcification *in vitro*

**FIGURE 5:**
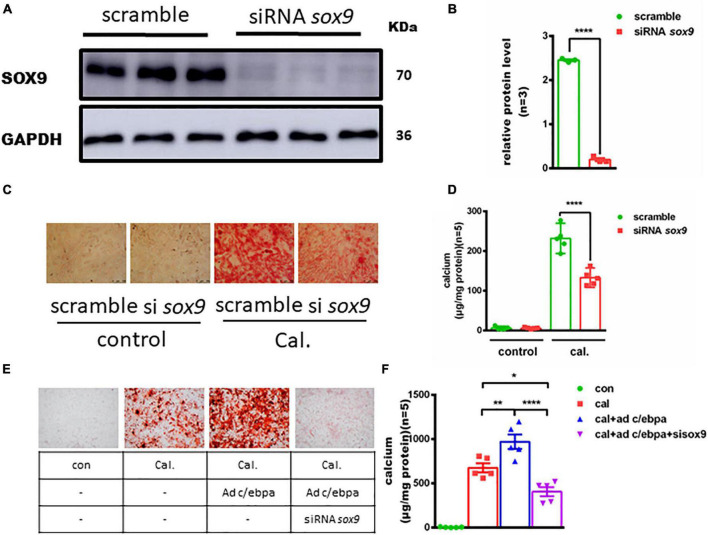
Silencing of the Sox9 inhibits the calcium-/phosphate-induced vascular calcification. **(A)** Western blotting analysis of VSMCs vascular calcification for SOX9 proteins. **(B)** Semiquantitative analysis of Western blotting for SOX9 protein expression (*n* = 3). **(C)** Representative images of alizarin red staining confirmed calcium deposition in VSMCs for the indicated experimental groups. Typical areas at 10 × magnifications are placed beside the respective panels. **(D)** Quantitative calcium assay for VSMCs for the indicated experimental groups (*n* = 5). **(E)** Representative images of alizarin red staining confirmed calcium deposition decreased in VSMCs incubated with Ad-c/ebpα and siRNA *sox9* in calcium/phosphate medium. Typical areas at 10 × magnifications are placed beside the respective panels. **(F)** Quantitative calcium assay for VSMCs for the indicated experimental groups (*n* = 5). *p < 0.05, **p < 0.01, ****p < 0.0001.

To investigate the role of *sox9* in VSMC calcification, we silenced *Sox9* expression in VSMCs (Figures 5A,B). On the contrary, the depletion of *Sox9* significantly decreased the calcium deposition in calcium-/phosphate-treated VSMCs, as evidenced by alizarin red staining and calcium quantitative analysis (Figures 5C,D). To understand the regulation mechanism, we knock downed sox9 after overexpression of C/EBPα in VSMCs treated with calcium/phosphate and found an attenuated VSMC calcification (Figures 5E,F).

## Discussion

In this study, we reported the effect of the novel promotive calcification mediator C/ebpα on VSMCs under the calcium/phosphate treatment condition. C/ebpα mRNA and protein levels were upregulated in calcium-/phosphate-induced VSMCs calcification *in vitro* and vitamin D-injected murine model *in vivo*. Besides, knockdown or overexpression of C/ebpα expression reduced or increased calcium-/phosphate-induced calcium deposition in VSMCs. Mechanistically, knockdown of C/ebpα attenuated VSMC calcification *in vitro* through downregulation of osteogenic transcription factors, *Bmp2* and *Sox9*, and upregulation of *Sma* and *Opn*. This study offers a new insight into the role of C/ebpα in CVD and provides sufficient evidence to confirm the promotive effect of C/ebpα on vascular calcification by mediating SOX9 expression.

It is well known that vascular calcification is prevalent in patients with CKD and diabetes. In these patient population, medial arterial calcification, which is located mainly in tunica media that contains VSMCs and elastic tissues, represents the specific change that is independent from AS (Chen et al., 2020). Recently, researchers come to the consensus that VSMCs can maintain different phenotypes, with osteoblasts, chondrocytes, adipocytes, and macrophage foam cells being the featured cell types, typically, the change from contractile to chondrogenic phenotype for the characteristic of developing vascular calcification (Durham et al., 2018). In response to the vessel plasticity, VSMCs are characterized by the expression of SMCs, i.e., specific contractile proteins, such as *Sma*, *Cnn1*, *Myh11*, *Col1a1*, and *Fn1*, all of which are confirmed in our model (Supplementary Figure 3). Similar to osteogenic differentiation of bone, vascular calcification is also characterized by key osteogenic regulators, including Col1, matrix Gla protein (MGP), OPN, MMP, BMP2, and the master osteogenic transcription factor, Runx2, and decreased expression of VSMCs marker simultaneously (Demer and Tintut, 2008; Chen et al., 2020). Accordingly, our experiments showed the successful construction of calcification model both *in vitro* and *in vivo*. We observed the significant upregulation of osteogenic genes and downregulation of SMA.

The C/ebpα was reported as one of the adipocyte markers during the adipogenic differentiation of VSMCs (Davies et al., 2005). Zhou et al. (2015) demonstrated that CHOP deficiency in aortic VSMCs attenuated the atherosclerotic plaque in *Chop*^fl/fl^*SM22*α*-CreKI^+^Apoe*^–/–^ mice treated with Western diet through reducing proliferation. Yue Liu et al. also demonstrated that cortistatin inhibited the osteogenic differentiation of VSMCs by decreasing the expression of CHOP (Liu et al., 2016). As one of the CHOP families, this is the first article to report the association of C/ebpα in medial vessel calcification according to our best acknowledgment. Recently, it is confirmed that the activation of adipogenic transcription promotes the differentiation of osteoclast precursors into mature osteoclasts, which disturbs calcium homeostasis (Muruganandan et al., 2020). Furthermore, Malgorzata Furmanik et al. demonstrated that endoplasmic reticulum (ER) stress played a key role in vascular calcification, and they reported the robust association between CHOP and vascular calcification (Furmanik et al., 2020). Previous studies have demonstrated the role of C/EBPα in mediating osteogenic genes, such as ALP, BMP2, or MSX2 (Ichida et al., 2004; Fan et al., 2009; Casado-Diaz et al., 2016). In contrast to previous studies, we observed that BMP2 and SOX9 were changed at mRNA and protein levels after silencing C/ebpα in the *in vitro* calcification model. Interestingly, SMA was upregulated by silencing C/ebpα. A previous study also demonstrated that the C/EBP family has a promotive effect on airway SMCs calcification (Ambhore et al., 2018). Theoretically, the imbalance of C/EBP isoform expression rather than C/ebpα alone promotes airway SMCs calcification (Borger et al., 2002). However, there was no difference in CEBP family expression in the VSMC calcification model, except for C/ebpα (Supplementary Figure 4).

The SOX9 is a transcription factor belonging to the SRY family which was proved to regulate chondrocyte differentiation. Hattori et al. (2010) demonstrated that downregulation of SOX9 was essential for endochondral ossification. Different from the mechanism in endochondral ossification, SOX9 is considered as a key regulator for smooth muscle differentiation. The SOX9-dependent pathway was confirmed to be essential in the TNF-α-induced downregulation of VSMCs contractile genes and the increases in cell proliferation and migration (Yu et al., 2018). Upregulation of SOX9 expression plays a key role in the VSMCs phenotype transdifferentiation and calcification deposition during plaque development (Augstein et al., 2018). Accordingly, this research confirmed SOX9 upregulation in vascular calcification by silencing SOX9 expression. In addition, previous research has reported the connection between the expression of SOX9 and C/ebpα, which is similar to our results (Antoniou et al., 2009). Further studies are warranted to explore the detailed mechanism.

Some limitations should be noted in this study. First, the VSMCs were isolated from different murine descending aortas, which might be more sensitive to calcium/phosphate treatment. Second, the inhibitory or reversal effect of knockdown C/ebpα on VSMC calcification was not tested in knockout mice. Therefore, future studies using the knockout mice model or human samples are warranted to determine the precise role of C/ebpα in vascular calcification and to reveal the causal insight.

## Conclusion

This study demonstrated that C/ebpα contributes to vascular calcification in VSMCs induced by calcium/phosphate treatment. Mechanistically, C/ebpα promotes the calcium-/phosphate-induced VSMCs calcification *in vitro* and *in vivo* through upregulation of osteogenic gene SOX9. The results of this study indicate that C/ebpα may be a novel therapeutic target for vascular calcification.

## Data Availability Statement

The raw data supporting the conclusions of this article will be made available by the authors, without undue reservation.

## Ethics Statement

The animal study was reviewed and approved by the Experimental Animal Ethics Committee of Guangzhou Medical University.

## Author Contributions

PH, HY, and LJ designed and supervised the study. PC, WH, ZC, SW, and FG-M performed the study. HF, YL, YD, and BW managed the animals and agents. PC and WH wrote the manuscript. HY and PH revised the manuscript for important intellectual content. All authors contributed to the article and approved the submitted version.

## Conflict of Interest

The authors declare that the research was conducted in the absence of any commercial or financial relationships that could be construed as a potential conflict of interest.

## Publisher’s Note

All claims expressed in this article are solely those of the authors and do not necessarily represent those of their affiliated organizations, or those of the publisher, the editors and the reviewers. Any product that may be evaluated in this article, or claim that may be made by its manufacturer, is not guaranteed or endorsed by the publisher.
