# Supplementary material for: CCAAT/Enhancer-Binding Protein Alpha Is a Novel Regulator of Vascular Smooth Muscle Cell Osteochondrogenic Transition and Vascular Calcification
Source: Front Physiol. 2022 Feb 28;13:755371. doi: 10.3389/fphys.2022.755371 (PMC8918665; doi:10.3389/fphys.2022.755371)

**Table S1.** The primer sequences for quantitative RT-PCR

| Gene | Forward primer (5ʹ-3ʹ) | Reverse primer (5ʹ-3ʹ) |
| --- | --- | --- |
| *Runx2* | ACCATAACAGTCTTCACAAATCCT | CAGGCGATCAGAGAACAAACTA |
| *Alpl* | GGGACGAATCTCAGGGTACA | AGTAACTGGGGTCTCTCTCTTT |
| *Bmp2* | *GCTTCTTAGACGGACTGCGG* | GCAACACTAGAAGACAGCGGGT |
| *C/ebpα* | *ATGGCGTCCTCTCTGCTTG* | TGAAAGGTCAGCGTATGGCTT |
| *Sox9* | \| *AGTACCCGCATCTGCACAAC* \| \| --- \| | *ACGAAGGGTCTCTTCTCGCT* |
| *Opn* | *AATGCTGTGTCCTCTGAA* | *TCGTCATCATCATCGTCAT* |
| *Opg* | *GGGCGTTACCTGGAGATCG* | *CGTTGTCATGTGTTGCATTTCC* |
| *Sma* | *CCCAGACATCAGGGAGTAATGG* | *TCTATCGGATACTTCAGCGTCA* |
| *β-Actin* | *GTGACGTTGACATCCGTAAAGA* | *GCCGGACTCATCGTACTCC* |
| *Gapdh* | TGGATTTGGACGCATTGGTC | TTTGCACTGGTACGTGTTGAT |

**Figure S1**. Staining of smooth muscle cells in Confocal Microscopy.

DAPI/SMA/mix


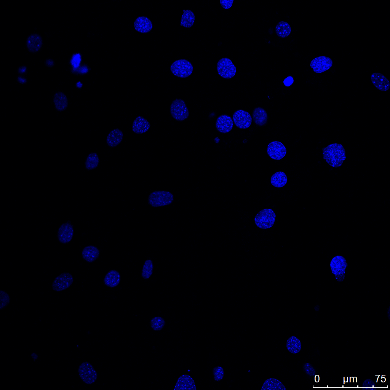

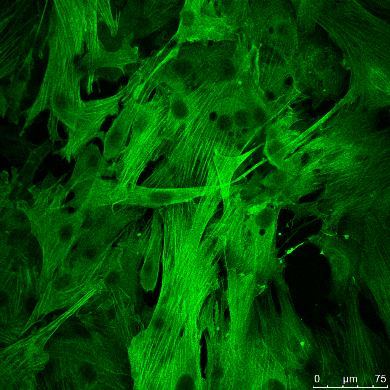

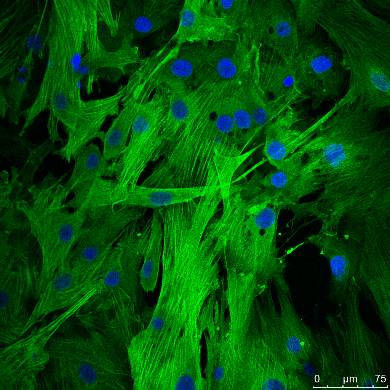


Figure S2. RT-qPCR of cell calcification on 4 days





Figure S3. mRNA expression of calcified VSMCs.


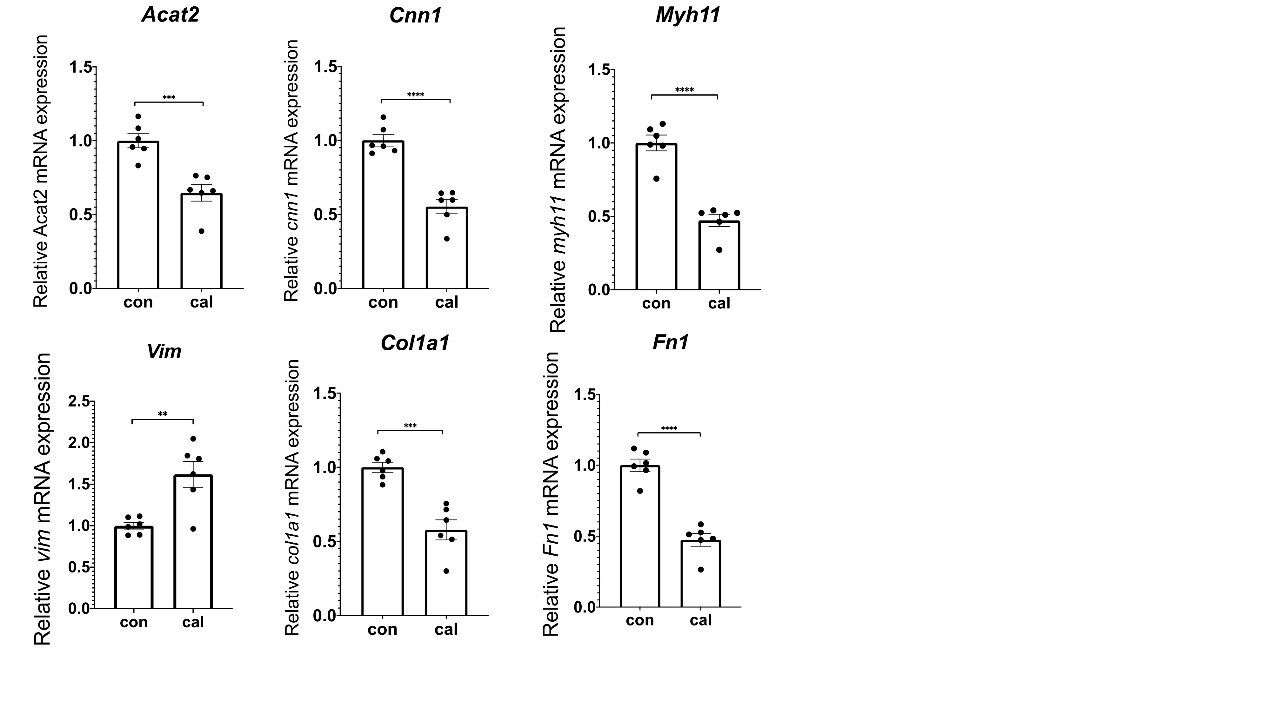


Figure S4 qPCR for CEBP family expression in VSMCs calcification.


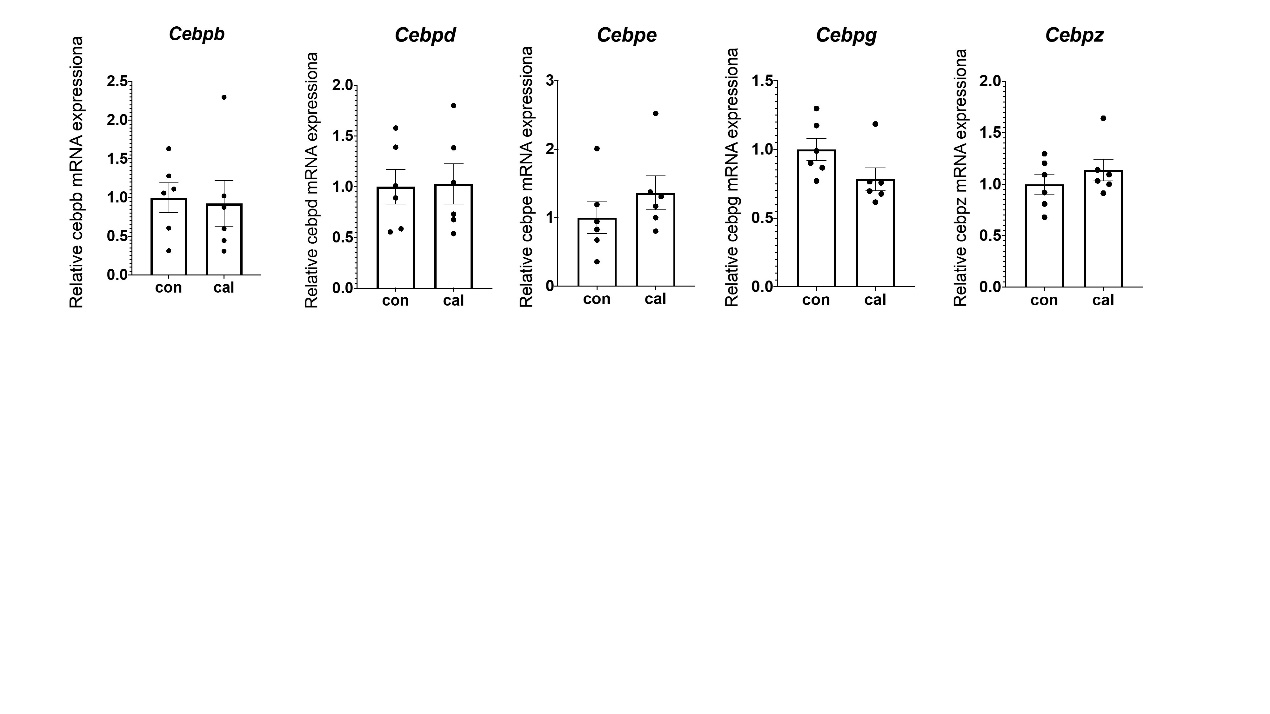


Figure S5 semi-quantification of Alizarin red staining of primary mouse VSMCs in this paper.













Figure S6 original plots of Western Blots


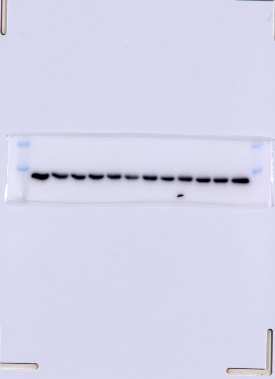

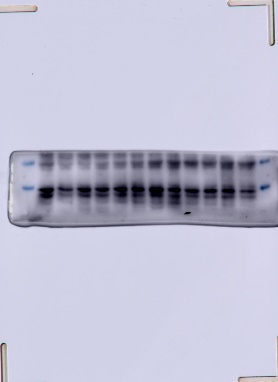

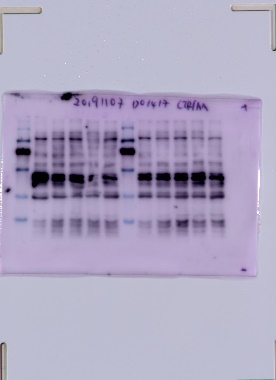

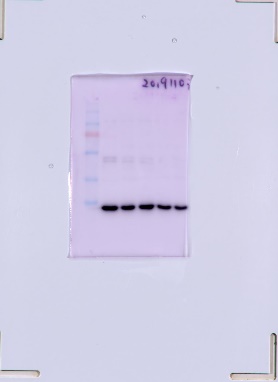


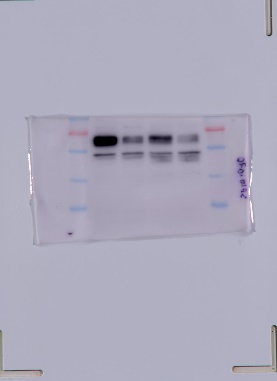

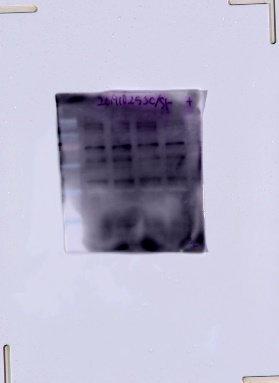

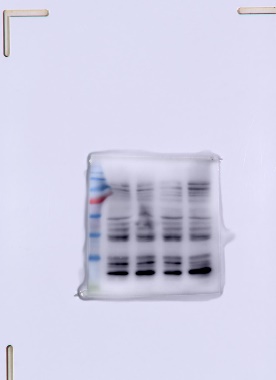

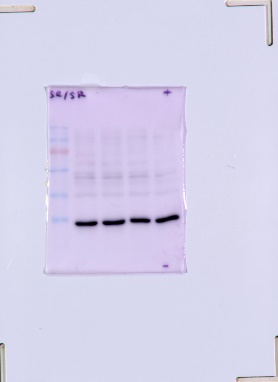


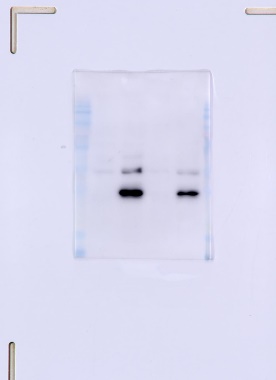

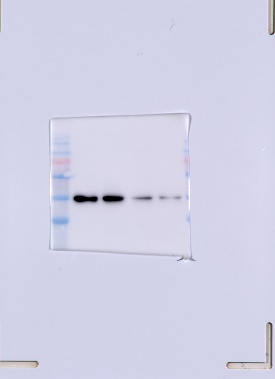

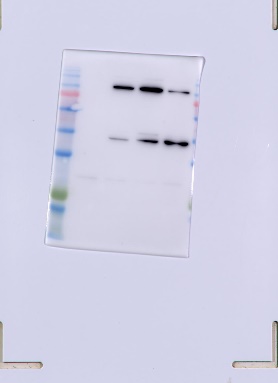

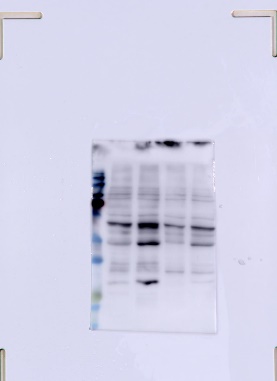


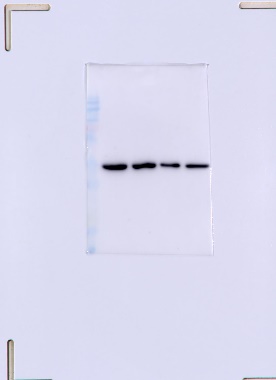

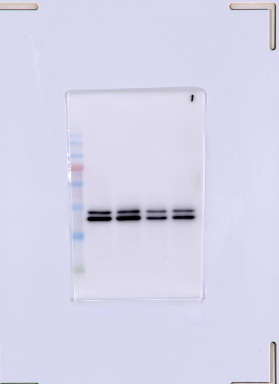

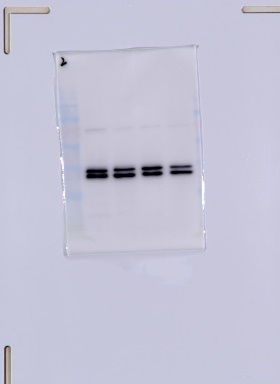

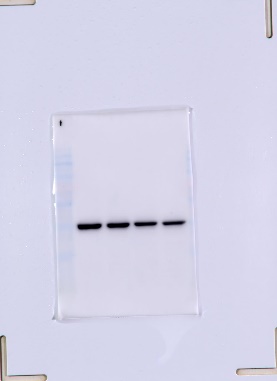

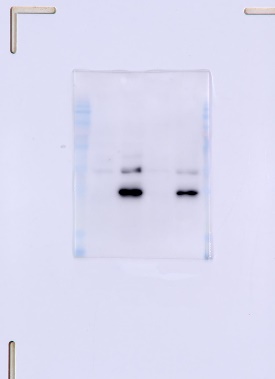

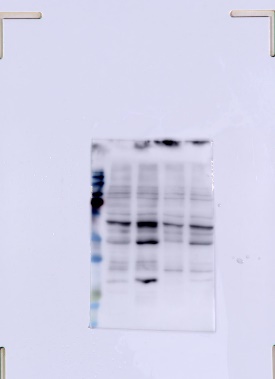

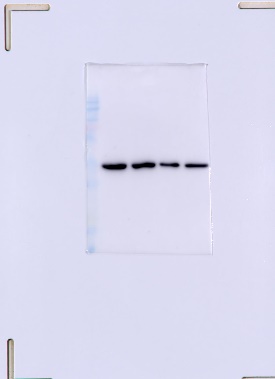

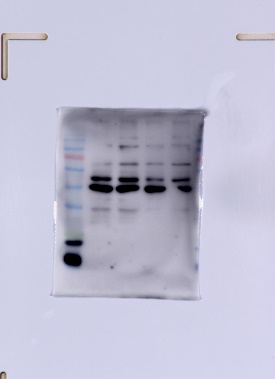

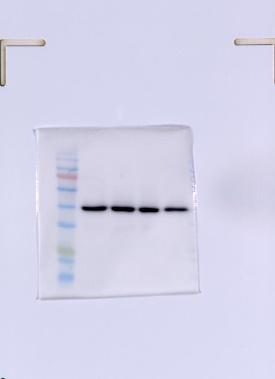

Supplement: Supplementary file 1 [file Data_Sheet_1.DOCX]
